# Affecting long-term fear memory formation through optical control of Rac1 GTPase and PAK activity in lateral amygdala

**DOI:** 10.1038/s41598-017-13674-9

**Published:** 2017-10-24

**Authors:** Aniruddha Das, Monica Dines, Jessica M. Alapin, Raphael Lamprecht

**Affiliations:** 1 Sagol Department of Neurobiology, Faculty of Natural Sciences,, Haifa, Israel; 2 The Integrated Brain and Behavior Research Center (IBBR), University of Haifa, Haifa, Israel

## Abstract

Fear conditioning, a behavioral model for studying fear-related disorders, is believed to be formed by alterations of synaptic efficacy mediated by changes in synaptic transmission and neuronal morphology in lateral amygdala (LA). Rac GTPase and its downstream effector p21-activated kinase (PAK) are involved in such key neuronal functions. Here we show that optical activation of Rac1 GTPase using photoactivatable form of Rac1 (PA-Rac1) in amygdala led to phosphorylation of PAK and inhibition of long-term but not short-term auditory fear conditioning memory formation. Activation of PA-Rac1 in LA one day after fear conditioning had no effect on long-term fear memory tested 24 hrs after PA-Rac1 activation. Inhibition of PAK in LA by microinjection of the PAK inhibitor IPA-3 30 minutes before fear conditioning enhanced long-term but not short-term fear memory formation. Our results demonstrate that photoactivation of Rac1 GTPase in lateral amygdala impairs fear memory formation. Moreover, Rac1 effector PAK activity during fear conditioning constrains the formation of fear memory in LA. Thus, Rac GTPase and PAK proteins may serve as targets for treatment of fear and anxiety disorders.

## Introduction

Experiencing a traumatic event may lead to the development of fear-related disorders. It is therefore important to understand the cellular and molecular mechanisms that regulate long-term fear memory formation and the ways to intervene by manipulating molecules to treat such disorders. While there are a number of experimental tools for studying fear and anxiety, one of the simplest and most straightforward is fear conditioning^1,2^. In fear conditioning an animal associates a neutral stimulus, such as a tone, with an aversive event, typically a mild footshock^3–7^. This paradigm is especially useful as a tool for studying the molecular basis of long-term fear memory because a putative site of memory, the lateral nucleus of the amygdala (LA), has been identified^3–10^. Thus, fear conditioning provides a behavioral tool and anatomical site to assess molecular mechanisms that might mediate changes in synaptic efficacy underlying long-term fear memory formation and fear-related disorders, such as posttraumatic stress disorder and phobias. Moreover, it serves as a behavioral model for studying new means for the treatment of such disorders and phobias.

Long-term fear memory (LTM) formation is believed to involve alterations of synaptic efficacy produced by modifications in neural transmission and/or structural modifications of synaptic connectivity within neuronal networks that subserve fear memory^10–12^. Rac1 GTPase and its downstream effector PAK have been shown to be involved in neuronal morphogenesis and synaptic transmission. Rac1 GTPase is a member of the Rho GTPases family molecular switches that cycle between an inactive GDP-bound state and an active GTP-bound form to regulate downstream effectors^13,14^. Rac1 is known to regulate proteins that affect actin polymerization and nucleation^15^. Rac1 can be activated following synaptic stimulation for example after NMDA receptor or Eph receptor activation^16^. Rac1 is involved in neuronal morphogenesis and is intimately involved in the regulation of dendritic spines structure and formation^13,15,17–19^. Moreover, Rac1 GTPase may facilitate or prevent axonal branching in different systems^20^. In addition, Rac1 is involved in regulating synaptic transmission. For example, Rac1 GTPase regulates the level of AMPA receptors in the synapse^21,22^. Rac1 is also involved in formation of synaptic plasticity including LTP and LTD, physiological models of memory^23^. Rac1 GTPase and PAK1 activities are affected by the mammalian target of rapamycin (mTOR) complex 2 (mTORC2) that was shown to be important for both L-LTP and auditory and contextual fear long-term memory formation^24^. Recently it was shown that activation of Rac1 in nucleus accumbens^25^ prevented the formation of a conditioned place preference to cocaine and activation of Rac1 in M1 cortex led to the shrinkage of dendritic spines and to the disruption of the acquired motor learning^26^. Thus, Rac1 activation in LA may affect fear memory formation and be a useful tool for intervening with fearful memory consolidation. Further evidence supporting this hypothesis are the observations that actin cytoskeleton dynamics in LA are needed for long-term fear memory formation^27–29^ and that changes in neuronal morphology in LA, known to be mediated by actin such as spine morphogenesis, are also involved in fear memory formation^30,31^.

We were therefore interested to study whether activation of Rac GTPase may affect fear conditioning memory formation. Toward that end, we employed a novel approach to photoactivate Rac1 GTPase in LA by light with high temporal and spatial resolution. In this photoactivatable Rac1 (PA-Rac1) a complete *Avena sativa* Phototropin1 LOV2-Jα domain (sequence 404–547) is conjugated to the N-terminus of a constitutively active Rac1^32^. LOV2 interacts with a C-terminal helical extension (Jα) in the dark and blocks binding of Rac1 to its effectors. Blue light (473 nm) induces unwinding of the Jα helix and releases steric inhibition, leading to Rac1 activation^32^. Activation of PA-Rac1 induces local phosphorylation of PAK^32^. Using this approach we explored whether activation of Rac1 GTPase in LA affects fear memory formation.

Rac1 exerts many of its effects through its downstream effector p21-activated kinase (PAK)^33^. Rac GTPase facilitates PAK phosphorylation and activation^32^. Active PAKs phosphorylate other proteins such as LIM kinase (LIMK) and myosin light chain kinase (MLCK), positively regulating actin dynamics and essential events in the process of neuroplasticity^34–36^. PAK is involved in neuronal morphogenesis and synaptic transmission and plasticity. For example, cortical neurons displayed fewer dendritic spines and an increased proportion of larger synapses in transgenic mice in which the catalytic activity of PAK is inhibited in the postnatal forebrain^37^. These mice had impaired bidirectional synaptic modifiability exhibiting enhanced LTP and reduced LTD in the cortex. The mice exhibited enhancement of AMPA receptor and possibly NMDA receptor mediated synaptic transmission. We therefore further investigated the roles of PAK in fear memory formation.

Thus, Rac1 and its downstream effector PAK have an effect on actin cytoskeleton leading to alterations in neuronal morphology and synaptic transmission. For example, Rac1 and PAK1 may affect downstream effectors such as LIMK and ROCK that further regulate actin-regulatory proteins such as cofilin, a F-actin binding, depolymerization and severing protein, leading in turn to changes in actin dynamics and spine morphogenesis^38^. Spine morphogenesis is intimately involved in fear conditioning in LA^30,31^. We were therefore interested to explore whether alteration of Rac1 or PAK activity in LA can affect fear LTM formation.

## Results

### Activation of Rac1 GTPase in LA leads to increase in PAK phosphorylation

**Figure 1:**
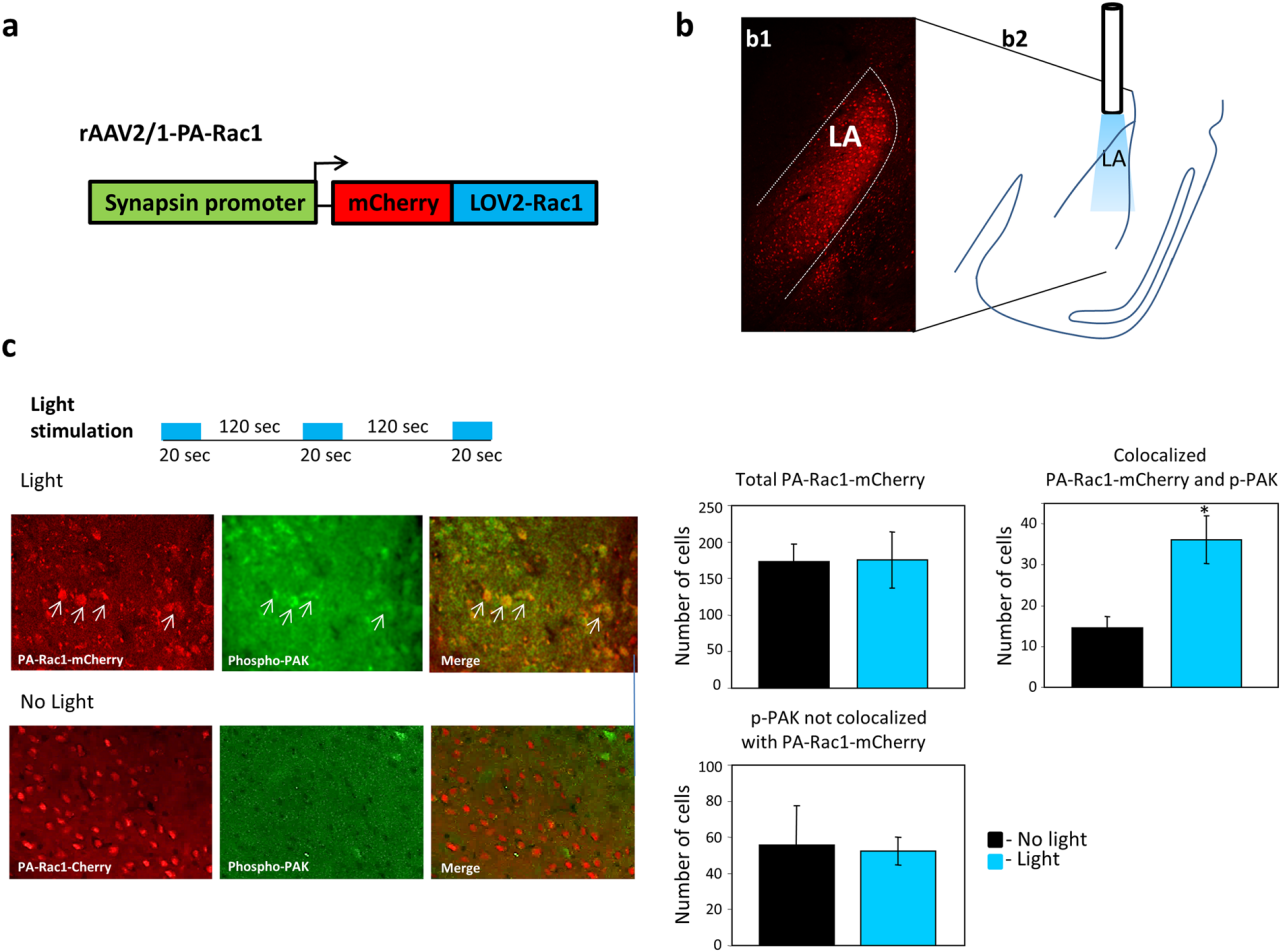
Activation of Rac1 GTPase in LA leads to increase in PAK phosphorylation. **(a)** Schematic description of the vector expressing Rac1 conjugated to LOV2 (PA-Rac1) and mCherry under the control of synapsin1 promoter. **(b)** PA-Rac1 is expressed in lateral amygdala (LA) as depicted by expression of mCherry (b1). The area of illumination by optic fiber includes the LA (b2). **(c)** Phospho-PAK is measured in the amygdala in light stimulated animals 30 minutes after stimulation (same light stimulating protocol used in behavior) and in animals that were not subjected to light. Left panels are representative immunohistochemistry of phospho-PAK and PA-Rac1-mCherry expression in AAV injected animals in light and no light animals. Arrows point at examples of phospho-PAK in PA-Rac1 expressing cells. Right graphs are quantifications of labeled cells (n = 6 mice no light, n = 7 light). The mean of total number of cells from slices from all animals in each group is shown. More phospho-PAK is detected in cells expressing PA-Rac (mCherry) in light exposed animals compared with animals that were not subjected to light (p < 0.02).

To study the effects of Rac1 GTPase activation in LA on fear memory formation we used the photoactivatable form of Rac1 (PA-Rac1). We expressed the PA-Rac1 under the control of synapsin promoter using AAV2/1 (Fig. 1a) in LA (Fig. 1b). We tested PA-Rac1 activation after light stimulation by measuring the level of phospho-PAK in mCherry-PA-Rac1 expressing cells. Control mice were injected with the AAV expressing the mCherry-PA-Rac1 but were not stimulated by light. Figure 1c shows a representative immunohistochemistry with phospho-PAK (green) in areas of PA-Rac1 expressing cells (red) in light and no-light groups (left panels). The right panels show graphs of the quantified results of total number of mCherry-PA-Rac1 labeled cells, the total phospho-PAK labeled cells that are not co-localized with mCherry-PA-Rac and number of cells that were labeled with both phospho-PAK and mCherry-PA-Rac1 detected 30 minutes after light stimulation in LA. We show that light stimulation leads to increased staining of phospho-PAK in cells in amygdala expressing the mCherry-PA-Rac1 compared to animals that were not subjected to light (p < 0.02). To test the effect of light only we implanted optic fibers in animals with no virus and shined light (n = 5). Controls were implanted with optic fibers but were not shined with light (n = 5). There is no significant difference between total phospho-PAK in LA between both groups (p > 0.7).

### Activation of Rac1 GTPase during training has no effect on short-term fear memory formation

**Figure 2:**
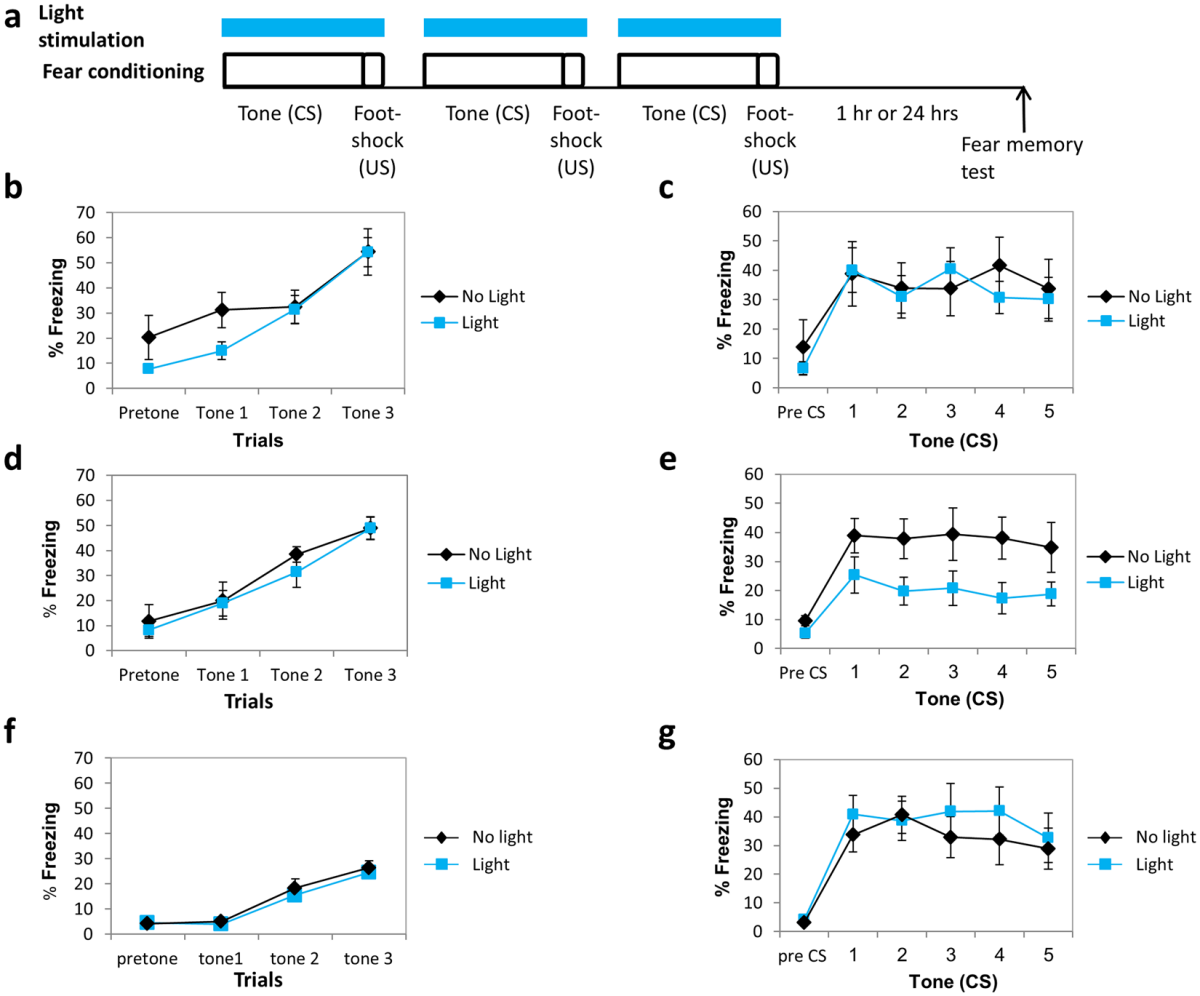
Activation of Rac1 GTPase in lateral amygdala impaired long-term but not short-term fear memory formation. **(a)** Description of the behavioral protocol. Mice injected with AAV containing PA-Rac1 were subjected to 3 tone-shock pairings. During each pairing the animals received blue light (473 nm) illumination in LA to activate the PA-Rac1. Fear memory was tested 1 hr (STM) or 24 hrs (LTM) after training. Controls were animals injected with AAV containing the PA-Rac1 and subjected to fear conditioning but without light. **(b)** Freezing throughout the tones presentations during training of animals used in the STM experiment subjected to light or no light control was analyzed. There is no significant difference between light and no light groups (F_(1,13)_ = 1.214, p > 0.2). **(c)** There was no significant difference between the light (n = 7) and no light (n = 8) groups when tested for fear STM (F_(1,13)_ = 0.029, p > 0.8). **(d)** Freezing throughout the tones presentations during training of animals used in the LTM experiment subjected to light or no light control was analyzed. There is no significant difference between light and no light groups (F_(1,18)_ = 0.422, p > 0.5). **(e)** Activation of Rac1 GTPase by light during fear conditioning (n = 10) inhibited fear LTM formation compared to the no light control group (n = 10) when tested 24 hrs after training (F_(1,18)_ = 4.671, p < 0.05). **(g)** Freezing throughout the tones presentations during training of animals that were not injected with virus but subjected to light or no light control that were used in the LTM experiment was analyzed. There is no significant difference between light and no light groups (F_(1,17)_ = 0.489, p > 0.4). **(f)** There is no significant difference between the animals that were not injected with AAV and subjected to light and animals that were not injected with AAV and not subjected to light (F_(1,17)_ = 0.305, p > 0.5) in the long-term fear conditioning memory test.

We were interested to explore the possibility that Rac1 GTPase activity in LA may affect fear short-term memory (STM) formation. Toward that end, we activated PA-Rac1 in LA bilaterally, using optic fibers, during the CS-US presentations in fear conditioning training (Fig. 2a). Control group expressed PA-Rac1 in LA and was subjected to fear conditioning training but not exposed to light stimulation through the optic fibers. Fear conditioning was tested 1 hr after training. Freezing during training was not different between the groups (F_(1,13)_ = 1.214, p > 0.2) there is no treatment × tone trial interaction (F_(2,26)_ = 0.864, p > 0.4) (Fig. 2b) indicating that the activation of Rac GTPase does not affect freezing per se, foot shock sensitivity and CS and US processing in LA. There was no significant difference between the light (n = 7) and no light (n = 8) groups when tested for fear STM (F_(1,13)_ = 0.029, p > 0.8) (Fig. 2c). There is no treatment × tone trial interaction (F_(2.455,31.909)_ = 1.032, p > 0.3) indicating that the rate of changes in fear responses along the trials was similar in all groups. These results show that Rac1 GTPase activation in LA has no effect on fear STM formation.

### Activation of Rac1 GTPase during training impairs long-term fear memory formation

We explored the possibility that Rac1 GTPase activity in LA may affect long-term fear memory formation. We activated PA-Rac1 in LA bilaterally, using optic fibers, during CS-US presentations in fear conditioning training (Fig. 2a). Control group expressed PA-Rac1 in LA and was subjected to fear conditioning training but not exposed to light stimulation through the optic fibers. Fear conditioning was tested 24 hrs after training. Freezing during tones presentation in training was not different between the groups (F_(1,18)_ = 0.422, p > 0.5), there is no treatment × tone trial interaction (F_(1.378,24.796)_ = 0.238, p > 0.7) (Fig. 2d). Activation of Rac1 GTPase during fear conditioning (n = 10) inhibited fear LTM compared to the no light control group (n = 10) when tested 24 hrs after training (F_(1,18)_ = 4.671, p < 0.05) (Fig. 2e). The treatment × tone trial interaction was not significant (F_(4,72)_ = 0.275 p > 0.8). To test the effect of light only on long-term fear memory we implanted optic fibers in animals with no virus and shined light during fear conditioning training as above (n = 10). Controls were implanted with optic fibers and trained for fear conditioning but with no light (n = 9). Freezing during tone presentation in training was not different between the groups (F_(1,17)_ = 0.489, p > 0.4), there is no treatment × tone trial interaction (F_(2,34)_ = 0.118, p > 0.8) (Fig. 2f). There is no significant difference between the groups (F_(1,17)_ = 0.305, p > 0.5) and no treatment × tone interaction (F_(4,68)_ = 1.004, p > 0.4) in the long-term memory test (Fig. 2g). Together these results show that activation of Rac1 GTPase during fear conditioning training inhibits fear LTM formation.

### Activation of Rac1 GTPase a day after training has no effect on long-term fear memory formation

**Figure 3:**
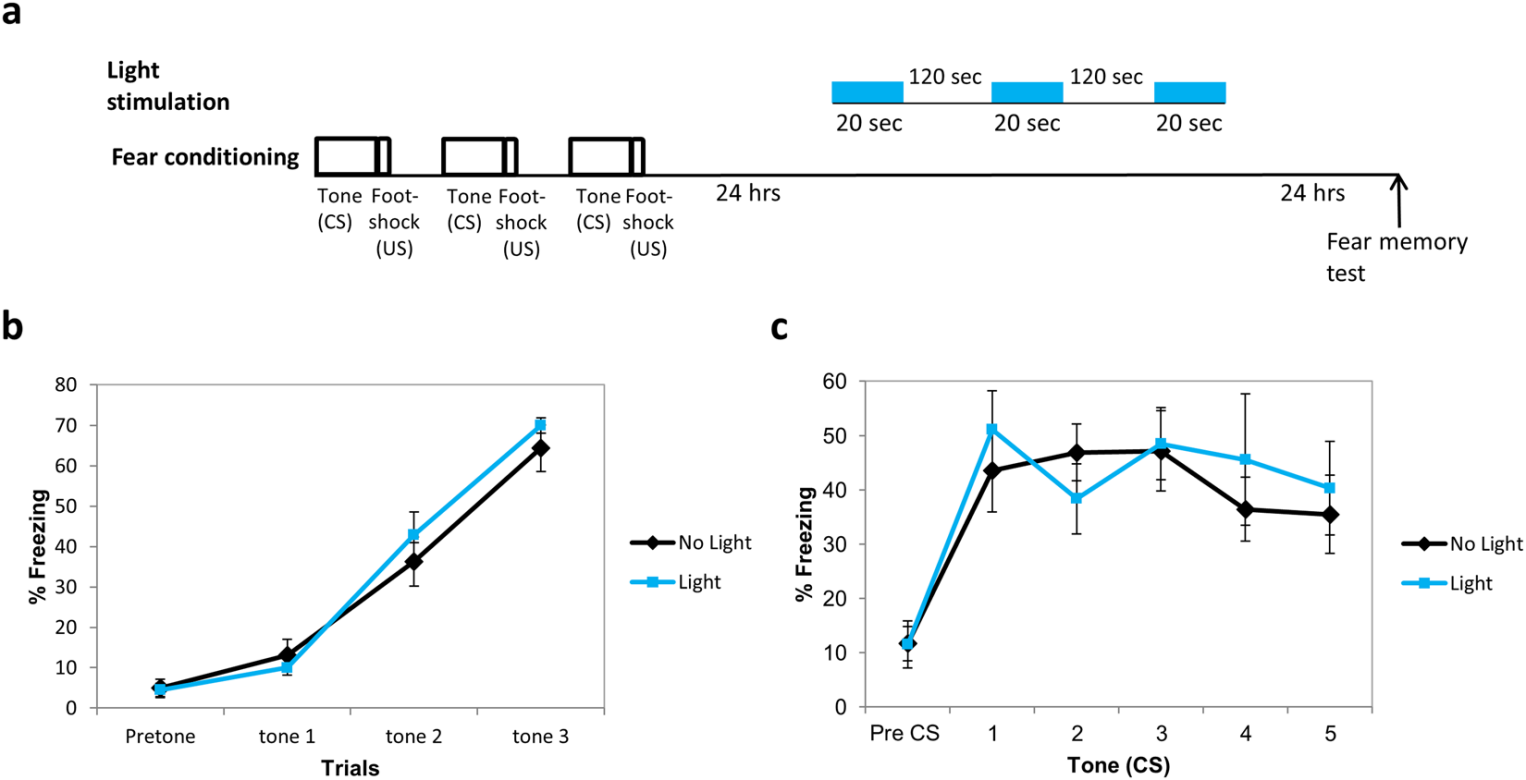
Activation of Rac1 GTPase a day after training has no effect on long-term fear memory formation. **(a)** Description of the behavioral protocol. Mice injected with AAV containing PA-Rac1 were trained for fear conditioning. Twenty four hours afterwards the mice were subjected to 3 blue light (473 nm) illumination pulses (20 seconds light stimulation with inter light stimuli interval of 120 seconds as above) in LA to activate the PA-Rac1. Fear memory was tested 24 hrs after light stimulation. Controls were animals injected with AAV containing the PA-Rac1 and subjected to fear conditioning but without light. **(b)** The groups were not different during fear training (F_(1,13)_ = 0.375, p > 0.5). **(c)** There was no difference between the Rac1 GTPase activated (n = 7) and non-activated groups (n = 8) in long-term fear memory (F_(1,13)_ = 0.104, p > 0.7).

The aforementioned results show that activation of the Rac1 GTPase during fear conditioning training impaired long-term fear memory formation. We were interested to study whether Rac1 GTPase activity has an effect on fear memory after the memory was consolidated. Toward that end we activated the PA-Rac1 in LA (same light intensity, duration and inter light stimuli intervals as above) 24 hrs after fear conditioning training and tested for long-term fear memory 24 hrs afterwards (Fig. 3a). Animals were not different in fear conditioning learning (F_(1,13)_ = 0.375, p > 0.5; no treatment × tone trial interaction F_(2,26)_ = 0.878, p > 0.4) (Fig. 3b). There was no difference between the Rac1 GTPase activated (n = 7) and non-activated groups (n = 8) in long-term fear memory (F_(1,13)_ = 0.104, p > 0.7) (Fig. 3c). The treatment × tone trial interaction was not significant (F_(4,52)_ = 1.107, p > 0.3). We conclude that Rac1 GTPase activation a day after fear conditioning has no effect on fear LTM.

### Inhibition of PAK activity in lateral amygdala has no effect on short-term fear memory

**Figure 4:**
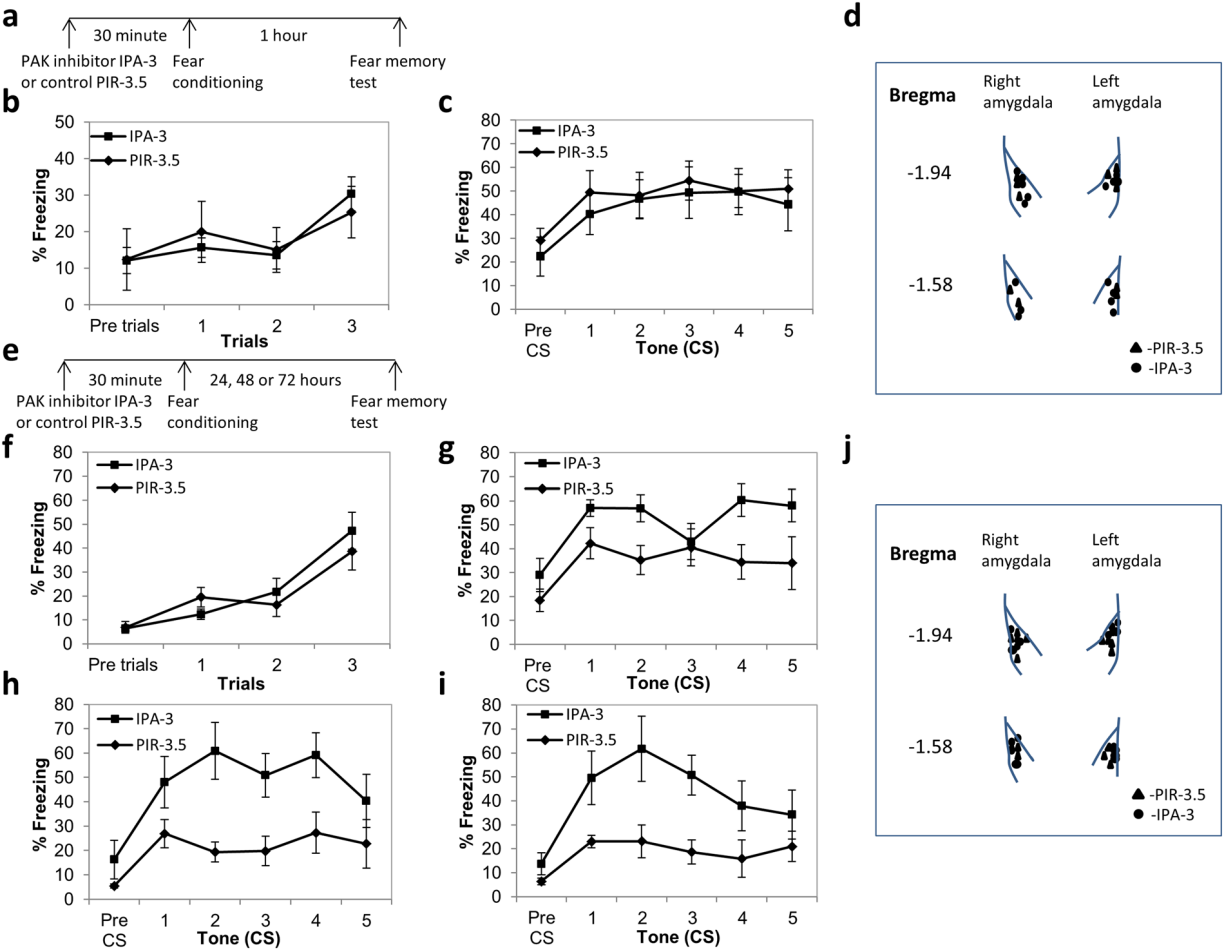
Inhibition of PAK activity in LA enhanced long-term, but not short-term, fear conditioning memory. **(a)** Schematic description of the short-term memory (STM) fear conditioning behavioral protocol. **(b)** Freezing during training was not different in animals to be tested for STM and microinjected with IPA-3 30 minutes before fear conditioning when compared to PIR-3.5 injected mice (F_(1,19)_ = 0.002, p > 0.9). **(c)** Microinjection of IPA-3 into LA 30 minutes before fear conditioning (n = 12) had no significant effect on fear memory compared to PIR-3.5 microinjected controls (n = 9) when tested 1 hr after training (F_(1,19)_ = 0.138, p > 0.7). **(d)** Cannulas placement in animals that participated in the STM test. **(e)** Schematic description of the long-term memory (LTM) fear conditioning behavioral protocol. **(f)** Freezing during training was not different in animals to be tested for LTM and microinjected with IPA-3 30 minutes before fear conditioning when compared to PIR-3.5 injected mice (F_(1,14)_ = 0.089, p > 0.7)**. (g)** Microinjection of IPA-3 into LA 30 minutes before fear conditioning enhanced fear memory compared to PIR-3.5 microinjected controls when tested 24 hrs after training (F_(1,14)_ = 4.885, p < 0.05) (n = 8 each). **(h)** Fear conditioning was enhanced in mice injected with IPA-3 into LA 30 minutes before training (n = 5) and tested 48 hrs after training when compared to PIR-3.5 microinjected mice (n = 6) (F_(1,9)_ = 7.674, p < 0.03). **(i)** Fear conditioning was enhanced in mice injected with IPA-3 into LA 30 minutes before training (n = 5) and tested 72 hrs after training when compared to PIR-3.5 microinjected mice (n = 6) (F_(1,9)_ = 6.812, p < 0.03). **(j)** Cannulas placement in animals that participated in the LTM test.

Rac GTPase is known to activate p21-activated kinase (PAK)^32,39^ (see Fig. 1). We were therefore interested to explore whether PAK in LA is involved in fear conditioning memory formation. Toward that end we used a specific PAK inhibitor IPA-3^40^. As a control we used the inactive PIR-3.5 which is a structural isomer of IPA-3^40^. We were interested to study whether PAK is involved in fear short-term memory (STM). Conditioned fear memory was assessed by measuring freezing responses elicited by the CS without the US 1 h after conditioning (STM). We microinjected IPA-3 into LA 30 min before fear conditioning and tested for STM (Fig. 4a). Control mice were microinjected with PIR-3.5 into LA 30 minutes before fear conditioning and tested for fear STM formation. Freezing during training was not different between the groups (F_(1,19)_ = 0.002, p > 0.9), there is no treatment × tone trial interaction (F_(2,38)_ = 0.942, p > 0.3) (Fig. 4b). There was no significant difference between the IPA-3 (n = 12) and PIR-3.5 (n = 9) microinjected groups when tested for fear STM (F_(1,19)_ = 0.138, p > 0.7) (Fig. 4c). There is no treatment × tone trial interaction (F_(4,76)_ = 0.511, p > 0.7). These results show that PAK in LA is not needed for fear STM formation.

### Inhibition of PAK activity in lateral amygdala enhanced long-term fear memory

We explored the possibility that PAK activity in LA may affect long-term fear memory formation. Conditioned fear memory was assessed by measuring freezing responses elicited by the CS without the US 24 h after conditioning (LTM). Freezing during training was not affected by microinjection of the IPA-3 into LA 30 minutes before fear conditioning (F_(1,14)_ = 0.089, p > 0.7) as well as the treatment × tone trial interaction during training (F_(2,28)_ = 0.2096, p > 0.14) (Fig. 4f). Microinjection of IPA-3 into LA 30 minutes before fear conditioning enhanced fear memory compared to the PIR-3.5 control when tested 24 hrs after training (F_(1,14)_ = 4.885, p < 0.05, n = 8 each) (Fig. 4g). The treatment × tone trial interaction was not significant (F_(4,56)_ = 1.445, p > 0.2). Fear conditioning was also enhanced in mice injected with IPA-3 (n = 5) and tested 48 hrs (F_(1,9)_ = 7.674, p < 0.03) (Fig. 4h) and 72 hrs (F_(1,9)_ = 6.812, p < 0.03) (Fig. 4i) after training when compared to animals injected with PIR-3.5 (n = 6). There was no treatment × tone trial interaction (48 hrs- F_(4,36)_ = 1.068, p > 0.3; 72 hrs- F_(4,36)_ = 1.664, p > 0.17). These results show that the effect of PAK inhibition during training on fear memory lasts over long period of time. There was no significant difference in fear LTM between animals injected into LA with the PIR-3.5 (n = 4) and animals injected with vehicle (n = 5) (F_(1,7)_ = 1.412, p > 0.2). There was no treatment × tone trial interaction (F_(4,28)_ = 0.473, p > 0.7). Cumulatively, the results show that PAK activation constrains fear memory formation and that its inhibition leads to enhancement of fear LTM.

## Discussion

In this study we examined whether Rac GTPase activation and PAK inhibition affect short- and long-term fear memory formation in lateral amygdala. Toward that end, we used a novel optogenetic approach to activate Rac GTPase in LA and advanced pharmacology to inhibit PAK activity in LA. We revealed that activation of Rac1 GTPase in LA reduced fear LTM and inhibition of PAK in LA enhanced fear LTM. We therefore conclude that activation of Rac1 GTPase during fear conditioning impairs fear LTM formation and that PAK activities in LA during fear conditioning constrain the creation of fear LTM. Thus, manipulating Rac GTPase and PAK activities in LA during fear conditioning determine the intensity of fearful memories.

To study whether activation of Rac1 can affect fear LTM formation we used an advanced optogenetic technique to activate Rac1 GTPase in lateral amygdala transiently and to explore the effects on long-term memory. Using photoactivatable Rac1 GTPase we revealed that activation of Rac1 in LA inhibited long-term but not short-term fear memory formation. These observations imply that increase of Rac GTPase activity during training does not impair fear memory acquisition but rather its consolidation. Furthermore studies have shown that fear conditioning training leads to specific response to the conditioned tone during STM and LTM tests without generalization to the control (not conditioned tone) showing that measuring freezing represents a memory specific test and not a generalization or non-specific effects^41^. Thus, activation of Rac GTPase can be useful for controlling the formation of pathological long-term memories in LA.

These results also demonstrate that the level of Rac1 activity in amygdala is critical for the ability to form memory. A study has shown that Rac activity presumably at lower level, as no additional activation during learning or afterwards was applied, can contribute the formation to both STM and LTM^42^ whereas we show that high activity of Rac1 inhibits specifically fear LTM. Thus, one conclusion that may be drawn is that individuals with high basal level of Rac1 activity, or at the time of learning, are more resistant for the formation of long-term fear memories. Consistent with this conclusion is a study showing that mice deficient for breakpoint cluster region (BCR) or BCR-related (ABR) Rac GTPase-activating proteins exhibit enhanced basal Rac1 activity and impaired spatial and object recognition memory^43^.

The photoactivation of Rac1 method was used recently *in vivo* in other studies. Light activation of PA-Rac1 in nucleus accumbens during cocaine pairing prevented the formation of a conditioned place preference to cocaine tested a day after conditioning^25^. In another study, an optoprobe, AS-PaRac1 (activated synapse targeting photoactivatable Rac1), that can label recently potentiated spines specifically and induces the selective shrinkage of the AS-PaRac1-containing spines in motor cortex upon activation, was used. AS-PaRac1 activation immediately after or a day after, but not two days after, training for motor learning task (walking on a rod) led to the disruption of the memory for the acquired motor task^26^. We show impairment in fear memory when PA-Rac1 is activated during training but not a day afterwards. The differences in time frames of Rac1 involvement could stem from the different behavioral tasks and brain regions that subserve them (fear conditioning and amygdala and motor task and cortex).

It should be noted that in culture neurons light activation of LOV2-Rac1 led to inhibition RhoA for at least 2 minutes following light stimulation^32^. This suggests that PA-Rac1 activity may affect Rho GTPase and its downstream effectors functions. Rho-associated protein kinase (ROCK) activity was shown to be needed in fear memory formation in LA^44^. In addition it implies, together with the observation that PA-Rac increases PAK phosphorylation minutes following its activation, that the effect of PA-Rac on cell signaling in LA exceeds the time window of stimulation during CS-US presentation.

Cumulatively, activation of Rac1 GTPase is being established as a novel and exciting mean for interrupting with various types of memories formation in different brain regions. This could be useful for treatment of harmful memories related to trauma and addiction.

Rac1 GTPase affects PAK activity^39^ and activation of PA-Rac1 induces PAK phosphorylation^32^ (Fig. 1 in our study). We were therefore further interested to study whether PAK is involved in LA in fear memory formation. We show that microinjection of PAK inhibitor IPA-3 into LA enhanced fear long-term memory formation. Microinjection of the PAK inhibitor IPA-3 into LA before fear conditioning had no effect on short-term fear conditioning memory. These results show that PAK activity in LA suppresses the consolidation of short-term memory into long-term fear memory. These observations also imply that PAK activity is not involved in fear memory acquisition.

Other studies exploring the roles of PAK in brain functions include genetically modified mice where alteration of PAK activity is induced over long period of time and in whole forebrain or brain. Interfering with PAK activity over long period of time and in large brain regions may lead to long-lasting neuronal alterations in these brain regions and different results than ours. For example, transgenic mice expressing dominant negative form of PAK exhibited impaired consolidation/retention of spatial (Morris water maze) and contextual fear conditioning memories^36^. However, auditory fear conditioning memory was found to be intact in these mice.

Interestingly, a recent study shows a role for PAK in amygdala in a social behavior task where the time exploring a novel mouse and previously familiar mouse is compared. Preinjection of the inhibitor IPA-3, but not the inactive isomer PIR-3.5, into the BLA 30 minutes before training leads to novel mouse preference and indicates of an enhanced social preference^45^.

Together our results show that Rac1 and PAK activities affect fear memory formation in LA. Extensive evidence from previous studies show that these proteins are involved in cellular processes that may affect synaptic efficacy and the communication between neurons required for memory formation^11,13–17^. These changes include alteration in synaptic connectivity between neurons, changes in postsynaptic morphology especially of dendritic spines and alteration in excitatory synaptic transmission by affecting, for example, the level of glutamate receptors at the synapse.

In this study we show for the first time that Rac1 GTPase and PAK activities in LA constrain long-term, but not short-term, fear memory formation. Thus, the level of Rac GTPase and PAK activity in LA during a fearful experience can determine whether it will be consolidated into long-term memory or not. Modulating the activity of these proteins may serve to control fear and anxiety and may be targeted for treatment of fear-related disorders.

## Materials and Methods

### Animals

Male C57BL6 mice (8–10 weeks, 24–27 grams) were used in this study (Harlan Laboratories). Following surgery mice were housed separately at 22 ± 2 °C in a 12 h light/dark cycle, with ad libitum access to food and water. All experiments were done following the instructions and approval of University of Haifa animal ethics committee for animal experiments observing National Institutes of Health guidelines and all experiments were performed in accordance with the relevant guidelines and regulations.

### Surgical procedures

Animals were anesthetized with Medetomidine (Domitor) 1 mg/ml and Ketamine 100 mg/ml cocktail, diluted in sterile isotonic saline (administered doses: Ketamine 50 mg/kg; Domitor 0.5 mg/kg; 100 µl/10gm of animal body weight). Dipyrone 50% was injected for analgesia before surgery and consecutive 3 days after surgery. Guide stainless-steel cannulas (23 gauge) were implanted bilaterally by stereotaxic surgery (Neurostar stereo drive) 1.5 mm above the LA. After surgery animals received antibiotics Baytril (5 mg/kg; Enrofloxacin). Antibiotics were applied for 3 consecutive days after surgery. The animals recovered for at least 7 days before the behavioral training.

### Microinjection of PAK inhibitor (IPA3) and control drug (PIR-3.5)

Group I p21-activated kinase (PAK) inhibitor 1,1′-Dithiodi-2-napthol (IPA-3, Tocris bioscience) was injected into LA to inhibit PAK activity. The inactive isomer of IPA-3 6,6′-Dithiodi-2-napthol (PIR-3.5, Tocris bioscience) was used as control. Drugs were dissolved in saline and DMSO (1:1) at concentration of 500 µM. The injection cannula was connected via PE20 tubing, back filled with saline with a small air bubble separating the vehicle from the dissolved drug solution, to a 10 μl Hamilton micro-syringe, driven by a microinjection pump (CMA/100, Carnegie Medicine; or PHD 2000, Harvard Apparatus). Solution was injected at a rate of 0.5 μl/min. Total volume injected per amygdala was 0.5 μl. Following injection, the injection cannula was left for an additional 1 min before withdrawal to minimize dragging of injected liquid along the injection track.

### AAV virus production and injection

We used rAAV 2/1 vectors containing hSynapsin-PA-Rac1-mCherry (titer: 1.68E + 13VG/ml, Signagen Laboratories). PA-Rac1-mCherry was obtained from Addgene. Animals were anesthetized following the procedure mentioned above. AAV was injected (1 µl/hemisphere, 0.1 µl/min) into LA/BLA following a stereotaxic surgery (Neurostar stereo drive). After virus injection, intracranial optic fiber (Thor labs, Fiber Optic Cannula, Ø1.25 mm Stainless Ferrule, Ø200 µm Core, 0.39 NA) was implanted on the same line and 0.5 mm above the virus injection place. Animals were allowed to recuperate for 4 weeks before behavioral experiments.

### Fear conditioning

With drug injected animals: Mice were habituated in the training Plexiglas conditioning chamber (Coulbourn Instruments) for 10 minutes. On the next day the animals were subjected to the fear conditioning protocol. Animals were placed in the conditioning chamber (Pre-tone) and two minutes afterwards subjected to three pairs of tone (Conditioned stimulus (CS) - 30 secs, 2.8 kHz, 85 dB) that co-terminated with a foot shock (Unconditioned stimulus (US) - 2 secs, 0.8 mA). The inter-trial interval was 120 secs. Mice were tested in a different context 1 hour after training for short-term memory or 24 hours 48 hours or 72 hours after training for long-term memory.

With virus infected animals: Optogenetic protocol: On the day of training, mice were placed in a training chamber (Coulbourn Instruments). Mice were allowed to acclimate with the chamber for 2 mins and then subjected with 3 pairs of tone (Conditioned stimulus (CS) - 20 secs, 2.8 kHz, 85 dB) that co-terminated with a foot shock (Unconditioned stimulus (US) - 2 secs, 0.8 mA). The inter trial interval was 120 secs. A blue light (473 nm) from a laser was ON during all the CS-US pairing (20 secs) to stimulate the photoactivatable Rac1. Optic fibers were connected to a 473-nm blue laser diode (Shanghai Dreamlasers) via a FC/PC adaptor. The light intensity ~15 mW/mm^2^ was measured at the tip of the fiber. A control group of animals got equal amount of virus microinjection into LA/BLA along with the fiber optic implantation but did not receive light stimulation during the CS-US pairings. Mice were tested in a different context 1 hour after training for short-term memory or 24 hours after training for long-term memory.

Behavior was recorded and the video images were transferred to a computer equipped with an analysis program (FreezeFrame). The percentage of changed pixels between two adjacent 0.25 s images was used as a measure of activity.

### Immunohistochemistry

One day after the behavioral testing the animals were subjected to the light stimulation as above. Thirty minutes after stimulation mice were anesthetized and perfused intracardially with 50 ml of PBS (pH = 7.4) followed by 50 ml of 4% paraformaldehyde (Sigma Aldrich) in PBS using a peristaltic pump (easy-load Master flex, Cole Parmer, model no: 7518-00, U.S.A.). Brains were removed and immersed in PBS containing 30% sucrose and 1% PFA for 48 hours for post fixation of the tissue and then stored at −80 °C until slicing. Brain slices were prepared (40 μm thickness) using microtome (LEICA SM 2000R) and kept floating in PBS. Slices were permeabilized with 0.2% Triton in PBS for 15 minutes, then blocked (in room temperature for 1.5 hours) with 5% normal goat serum (NGS) prepared in 0.1% Triton in PBS. These slices were probed with anti-phospho-PAK (Phospho-Thr423/402/421, 1:200, Novus Biologicals) in 1% NGS and 0.1% Triton in PBS. All slices were probed by the primary antibodies for overnight at 4 °C. Slices were then washed thrice with PBS and subjected to the secondary antibody Alexa Fluor 488 anti-rabbit (1:500, in PBS, Invitrogen, ThermoFisher Scientific) for 1.5 hrs at room temperature followed by 3 washes with PBS before mounting. Slices were mounted on microscope slides with slow fade Gold antifade reagent (ThermoFisher Scientific) to minimize the fluorescence quenching. The slices from the animals were mounted on slides. The slices were visualized and LA/BLA areas were photographed. The images of random photographs were analyzed with Imaris or Image J software to count the number of cells on a brain slice. First, parameters were set to count the number of cells with mCherry expression. We covered all visible red cells on a slice within the LA/BLA. We followed the same procedure with green filter separately (for p-PAK staining) to count cells in the same area where mCherry was monitored.

### Histology

After behavioral testing of animals injected with IPA-3 inhibitor or PIR-3.5 was completed animals were sacrificed and brains were quickly removed, placed on dry ice and stored at −80 °C until use. Brains were sliced (40 μm) and stained with cresyl violet acetate to verify the cannula placements within LA/BLA using bright field microscope (LMD 7000; Leica). Only mice with cannula tips at or within the boundaries of the LA/BLA were included in the data analysis. Mice injected with AAV were perfused as above and tested for expression of mCherry in brain. Only mice with expression of mCherry in LA/BLA were included in the data analysis.

### Statistics

Data were analyzed with repeated measures ANOVA for behavioral studies and t-test for the immunohistochemistry study with an α level of 0.05 using the PASW statistics 20.
